# From resources to efficacy over time: a longitudinal study of psychological capital, emotion regulation, and teacher self-efficacy

**DOI:** 10.3389/fpsyg.2025.1764648

**Published:** 2026-01-13

**Authors:** Pu Zhang, Hong Zhang

**Affiliations:** 1 Faculty of Education, Sichuan Normal University, Chengdu, China; 2 College of Education, Fuyang Normal University, Fuyang, China; 3 School of Mathematical Sciences, Sichuan Normal University, Chengdu, China

**Keywords:** Chinese primary teachers, emotion regulation, longitudinal mediation, psychological capital, structural equation modeling, teacher self-efficacy

## Abstract

**Objective:**

The underlying mechanism through which teachers’ psychological capital (PsyCap) influences teacher self-efficacy (TSE) remains insufficiently understood. This study aims to examine the mediating role of emotion regulation (ER) in the association between PsyCap and TSE among Chinese primary school teachers using both cross-sectional and longitudinal data.

**Methods:**

A total of 606 Chinese primary school teachers were included at baseline. Data on PsyCap, ER, and TSE were collected. Structural equation modeling (SEM) was used to examine the cross-sectional mediation effect. In addition, a three-wave longitudinal subsample (*N* = 412) was analyzed using a time-lagged mediation model to test whether psychological capital predicts subsequent emotion regulation and, in turn, later teacher self-efficacy.

**Results:**

PsyCap, ER, and TSE were significantly correlated (*p* < 0.01). Cross-sectional SEM showed that PsyCap positively predicted ER (β = 0.63, *p* < 0.001) and TSE (β = 0.57, *p* < 0.001), and that ER positively predicted TSE (β = 0.33, *p* < 0.001). Bootstrapping confirmed a significant indirect effect of PsyCap on TSE via ER (β = 0.21, *p* < 0.001), indicating partial mediation (total effect β = 0.78, *p* < 0.001). Longitudinal time-lagged analyses further supported the temporal ordering of effects: PsyCap at Time 1 predicted ER at Time 2 (β = 0.48, *p* < 0.001) and TSE at Time 3 (β = 0.29, *p* < 0.001), ER at Time 2 predicted TSE at Time 3 (β = 0.24, *p* < 0.01), and the indirect longitudinal effect was significant (β = 0.12, *p* < 0.01).

**Conclusion:**

There is a significant mediating effect of ER in the relationship between PsyCap and TSE among Chinese primary school teachers. Higher levels of PsyCap promote adaptive emotion regulation, which in turn enhances teachers’ self-efficacy. These findings provide longitudinal evidence for the psychological mechanisms underlying teacher self-efficacy and offer practical implications for professional development programs targeting teachers’ psychological capital and emotion regulation skills.

## 1 Introduction

Teachers’ psychological well-being and professional self-efficacy have long been recognized as pivotal determinants of educational quality in the Asia-Pacific region, where educational reforms increasingly emphasize both academic excellence and teacher well-being (Zee and Koomen, 2016). Teacher self-efficacy (TSE), defined as teachers’ beliefs in their capabilities to achieve desired instructional outcomes (Tschannen-Moran and Hoy, 2001), has been consistently linked to effective pedagogy, student motivation, and positive classroom climate (Burić and Kim, 2020; Jerrim et al., 2023). Meta-analytic evidence further indicates that TSE is a robust predictor of classroom processes, student engagement, and teachers’ psychological health (Zee and Koomen, 2016).

Beyond its direct association with instructional quality, TSE influences classroom outcomes through multiple mechanisms, including teachers’ instructional persistence and adaptive strategy use, as well as classroom management behaviors and emotional engagement with students (Duan and Zhao, 2024). Teachers with higher TSE are more likely to implement student-centered practices, respond constructively to classroom challenges, and sustain positive emotional climates, which in turn foster student motivation and engagement, particularly in primary school settings (Ma et al., 2025). However, existing evidence remains largely based on cross-sectional studies (Burić and Macuka, 2018; Klassen and Chiu, 2010; Li, 2023), providing limited understanding of how teachers’ efficacy beliefs develop over time within the dynamic sociocultural environments of Asia-Pacific schooling.

Although psychological capital, emotion regulation, and teacher self-efficacy are theoretically related and empirically correlated, they represent conceptually distinct constructs operating at different functional levels. Psychological capital reflects a set of relatively stable psychological resources that teachers draw upon when facing professional challenges (Luthans et al., 2007). Emotion regulation, by contrast, refers to dynamic regulatory processes through which individuals monitor, modify, and manage emotional experiences in situational contexts (Gross, 1998). Teacher self-efficacy represents an outcome-oriented belief system concerning teachers’ perceived capability to organize and execute instructional actions required to attain desired educational goals (Bandura, 1997; Tschannen-Moran and Hoy, 2001). Conceptually distinguishing psychological capital as a psychological resource, emotion regulation as a regulatory process, and teacher self-efficacy as an efficacy belief allows these constructs to be examined simultaneously without conceptual redundancy and provides a coherent basis for modeling a developmental pathway from psychological resources through regulatory processes to efficacy beliefs.

Within this evolving educational landscape, emotional processes play a central role in shaping teachers’ self-beliefs. Emotion regulation (ER)—the ability to monitor and adjust emotional responses to maintain effective functioning (Gross and John, 2003)—is a foundational mechanism that supports teachers’ resilience and instructional confidence (Brackett et al., 2010; Greenier et al., 2021). Teachers with strong ER capacities are more likely to manage stress, prevent burnout, and sustain positive interactions in emotionally demanding classrooms (Chang, 2020). Meanwhile, psychological capital (PsyCap)—a higher-order construct encompassing hope, efficacy, optimism, and resilience (Luthans et al., 2007)—represents a vital psychological resource that fuels professional engagement and well-being (Avey et al., 2011). In Asian educational systems, recent studies have begun to show that PsyCap enhances teachers’ adaptive coping, work engagement, and persistence (Gan and Cheng, 2021; Guo et al., 2022). Yet, empirical work examining how PsyCap and ER jointly contribute to TSE remains scarce, particularly in primary school settings and within the broader Asia-Pacific context. The resource–regulation–belief chain—linking psychological resources (PsyCap) to regulatory processes (ER) and self-efficacy beliefs (TSE)—has seldom been tested using longitudinal data, leaving temporal and developmental mechanisms underexplored.

The present study addresses these gaps by integrating PsyCap, ER, and TSE into a unified explanatory framework and empirically testing their direct and indirect relationships among Chinese primary school teachers. Specifically, this research contributes to the literature in three ways. First, it advances a theoretical model that conceptualizes the development of teacher self-efficacy as a function of teachers’ positive psychological resources and emotion-regulatory capacities, thereby enriching the resource–regulation–belief perspective in teacher psychology. Second, it employs both cross-sectional SEM and a three-wave time-lagged longitudinal design, using CFA/SEM and a time-lagged mediation model to examine whether psychological capital at an earlier time point predicts subsequent emotion regulation and, in turn, later teacher self-efficacy. Third, by focusing on Chinese primary teachers—a key yet underrepresented group in Asia-Pacific research—the study provides regionally grounded evidence that may inform teacher education and professional development initiatives across the region.

## 2 Literature review

### 2.1 Psychological capital

Psychological capital (PsyCap), comprising self-efficacy, hope, optimism, and resilience, has become central to teacher psychology and professional growth (Avey et al., 2011; Luthans et al., 2007). Within this framework, self-efficacy refers to teachers’ confidence in their ability to successfully perform instructional tasks and cope with professional demands; hope reflects goal-directed energy and the perceived capability to generate pathways toward desired teaching outcomes; optimism denotes a positive attributional style regarding present and future professional experiences; and resilience captures teachers’ capacity to recover and grow from setbacks and challenges encountered in educational contexts (Luthans et al., 2007).

A growing body of empirical research indicates that higher levels of PsyCap predict teachers’ well-being, work engagement, and mental health (Bertieaux et al., 2024; Gan and Cheng, 2021), and mediate key occupational outcomes such as job satisfaction, burnout, and motivation (Bidi et al., 2024; Hazan-Liran and Karni-Vizer, 2024; Ma, 2023). In both international and Chinese contexts, teachers with stronger PsyCap demonstrate greater emotional balance, adaptive coping, and occupational resilience (Guo et al., 2022; Liu and Du, 2024).

Moreover, PsyCap has been shown to promote organizational commitment, teacher retention, and professional satisfaction (Ekmekci et al., 2025; Zhang et al., 2025), and to be strengthened by contextual factors such as transformational and socially just leadership (Bellibaş et al., 2025). Meta-analytic evidence further confirms its systematic associations with motivation, academic achievement, and reduced burnout across educational settings (Li et al., 2023). Despite these advances, relatively few studies have examined the structural validity and underlying mechanisms of PsyCap among Chinese primary school teachers, particularly with regard to how specific PsyCap components shape instructional beliefs and emotion regulation processes. Taken together, these findings underscore PsyCap as a foundational psychological resource in education (Huppert, 2009), supporting the theoretical framework adopted in the present study.

### 2.2 Emotion regulation

Emotion regulation (ER) refers to processes through which individuals generate, monitor, and modify emotional responses (Gross, 1998; Thompson, 1994). In educational settings, ER is closely linked to teachers’ emotional labor and instructional functioning: antecedent-focused strategies are associated with higher job satisfaction and more adaptive teaching, whereas response-focused strategies are related to emotional exhaustion and burnout (Brackett et al., 2010; Lee et al., 2016; Taxer and Gross, 2018). Integrating the process model of ER with perspectives on teacher social–emotional competence (Jennings and Greenberg, 2009), recent syntheses indicate that teachers’ ER contributes to teaching effectiveness through improved instructional quality, enhanced teacher–student interactions, and reduced stress and burnout (Aldrup et al., 2024; Sutton and Wheatley, 2003).

Beyond correlational evidence, emerging intervention-based research provides stronger support for ER as a plausible mechanism. Reappraisal-focused approaches grounded in ER theory have been empirically tested in teacher-relevant contexts, demonstrating benefits for teacher well-being and classroom-related outcomes (Cohen et al., 2023). Similarly, brief experimental interventions using implementation-intention–based self-affirmation have shown short-term effects on teachers’ affective experiences and ER strategy use, suggesting that targeted regulation-oriented training can modify emotion-related processes (Morgan and Atkin, 2016). Moreover, mindfulness-based interventions—often theorized to operate partly via enhanced ER—have been synthesized in systematic reviews, and recent mediation-focused studies explicitly identify ER as a pathway through which such programs reduce stress and improve teacher well-being and classroom climate (Emerson et al., 2017; Rombouts et al., 2025).

Consistent with these findings, meta-analytic and large-scale evidence links teachers’ ER strategies to well-being and positive emotional outcomes, with adaptive strategies conferring benefits and maladaptive strategies elevating risk (Wang et al., 2023; Wang et al., 2025). In addition, theoretical and empirical syntheses suggest that teachers’ ER shapes classroom emotional tone and teacher–student relationships, indirectly influencing classroom climate and student adjustment (Aldrup et al., 2024; Jennings and Greenberg, 2009; Sutton and Wheatley, 2003). Nevertheless, longitudinal evidence explicitly testing ER as a temporal mediating mechanism in Chinese primary-school contexts remains limited. Accordingly, the present study conceptualizes ER as a mediating regulatory process linking teachers’ psychological resources (PsyCap) to subsequent efficacy beliefs (TSE), consistent with the proposed resource–regulation–belief framework.

### 2.3 Teacher self-efficacy

Teacher self-efficacy (TSE), grounded in Bandura (1997) social cognitive theory, reflects teachers’ beliefs in their capacity to influence student learning and effectively carry out instructional tasks (Armor, 1976; Gibson and Dembo, 1984). Early conceptualizations distinguished between general teaching efficacy, referring to beliefs about whether teaching can make a difference despite external constraints, and personal teaching efficacy, referring to beliefs about one’s own instructional competence. Building on this foundation, subsequent research has conceptualized TSE as a multidimensional construct extending across key domains of teaching practice, including instructional strategies, classroom management, and student engagement (Tschannen-Moran and Hoy, 2001, 2007). This multidimensional perspective captures the complexity of teachers’ professional beliefs and provides a robust framework for examining efficacy across diverse instructional contexts.

A substantial body of empirical research demonstrates that TSE is a critical predictor of both instructional processes and outcomes. Teachers with stronger efficacy beliefs tend to exhibit higher classroom quality, foster more supportive learning environments, and promote better student academic achievement and well-being (Caprara et al., 2006; Zee and Koomen, 2016). Beyond instructional effectiveness, TSE also functions as an important psychological resource for teachers themselves, buffering the negative effects of occupational stress and reducing vulnerability to burnout (Schwarzer and Hallum, 2008; Skaalvik and Skaalvik, 2007). This protective role is particularly evident among novice teachers, whose efficacy beliefs are still developing and are strongly shaped by mastery experiences, mentoring, leadership support, and collegial environments during the early stages of their careers (Day and Gu, 2013; Hoy and Spero, 2005; Hoy and Woolfolk, 1990; Weinstein, 1988).

More recent integrative research has begun to move beyond static views of TSE by examining the psychological and emotional mechanisms through which efficacy beliefs are formed and sustained. In particular, studies have linked psychological capital (PsyCap) and emotion regulation (ER) to teachers’ efficacy beliefs, suggesting that positive psychological resources facilitate adaptive emotional strategies, which in turn reinforce teachers’ confidence in managing instructional and relational demands (Klassen and Tze, 2014; Li, 2023; Luthans et al., 2007). However, within the context of Chinese primary education—where teaching is characterized by high emotional labor, intensive student care, and strong expectations for emotional display—empirical evidence remains limited regarding how PsyCap and ER jointly shape teachers’ efficacy beliefs. Clarifying this integrated mechanism is essential for advancing understanding of TSE development in emotionally demanding, non-Western educational contexts and for informing targeted professional development initiatives.

### 2.4 Present study

Drawing on positive psychology and social cognitive theory, the present study proposes a resource–regulation–belief framework to examine the relationships among psychological capital (PsyCap), emotion regulation (ER), and teacher self-efficacy (TSE). Specifically, the following hypotheses are tested:

*H1*: PsyCap is positively associated with TSE.

*H2*: PsyCap is positively associated with ER.

*H3*: ER is positively associated with TSE.

*H4*: ER mediates the relationship between PsyCap and TSE.

To further examine the temporal ordering of these associations, a three-wave longitudinal design is employed. It is hypothesized that PsyCap at Time 1 positively predicts ER at Time 2, which in turn positively predicts TSE at Time 3, thereby constituting a longitudinal mediation pathway.

## 3 Materials and methods

### 3.1 Participants

A total of 606 Chinese primary school teachers (Mage = 37.03 years, SD = 8.89) participated in the baseline survey (Time 1). Of these, 412 teachers completed all three waves of data collection and constituted the longitudinal sample, yielding an attrition rate of 32.0%. The baseline cross-sectional sample (*N* = 606) included 451 female teachers (74.4%) and 155 male teachers (25.6%). Detailed demographic characteristics of the participants are presented in Table 1. The longitudinal subsample (*N* = 412) showed a demographic distribution comparable to that of the baseline sample in terms of gender, age, and professional background. Cross-sectional analyses were conducted using the full baseline sample (*N* = 606), whereas all longitudinal analyses were based exclusively on the three-wave panel sample (*N* = 412).

**Table 1:** Demographic characteristics of the participants (*N* = 606).

Variable	Category	*n* (%)
Gender	Male	155 (25.58)
	Female	451 (74.42)
Age (years)	≤29	142 (23.43)
	30–39	247 (40.76)
	40–49	169 (27.89)
	≥50	48 (7.92)
Highest educational qualification	Associate degree	165 (27.23)
	Bachelor’s degree	419 (69.14)
	Master’s degree or above	22 (3.63)
Professional title	Unranked teacher	21 (3.47)
	Level 2 teacher	222 (36.63)
	Level 1 teacher	283 (46.70)
	Senior teacher	75 (12.38)
	Professor-level senior teacher	5 (0.83)
Current teaching stage	Lower grades (Grades 1–2)	225 (37.13)
	Middle grades (Grades 3–4)	199 (32.84)
	Upper grades (Grades 5–6)	182 (30.03)
Administrative role	None	202 (33.33)
	Homeroom teacher	346 (57.10)
	School-level administrator	58 (9.57)
School location	Provincial capital/first-tier city	95 (15.68)
	Prefecture-level city/county	339 (55.94)
	Township and rural areas	172 (28.38)
Employment status	Tenured teacher	508 (83.83)
	Contract/substitute teacher	98 (16.17)

### 3.2 Instruments

Three previously validated questionnaires were used to measure the core constructs of this study: Psychological Capital (PsyCap), Emotion Regulation (ER), and Teacher Self-Efficacy (TSE).

To assess Psychological Capital (PsyCap), the study adopted the Psychological Capital Questionnaire (PCQ-24) developed by Luthans et al. (2007), consisting of 24 items across four subdimensions: self-efficacy (SE1–SE6), hope (HOP7–HOP12), optimism (OPT13–OPT18), and resilience (RES19–RES24). Responses were rated on a 6-point Likert scale (1 = strongly disagree to 6 = strongly agree). The Cronbach’s α coefficients indicated high internal consistency across all subdimensions: self-efficacy α = 0.913, hope α = 0.916, optimism α = 0.910, and resilience α = 0.912. A representative item from the hope dimension was: “When facing challenging teaching goals, I can find multiple ways to accomplish them.” Although self-efficacy is included as a core component of psychological capital, it is conceptually distinct from teacher self-efficacy examined in the present study. Specifically, self-efficacy within PsyCap reflects a general positive psychological resource, whereas teacher self-efficacy represents a domain-specific belief regarding instructional capability. Consistent with prior research, these constructs were treated as related but theoretically distinguishable.

To measure Emotion Regulation (ER), the study employed the Emotion Regulation Questionnaire (ERQ-10) developed by Gross and John (2003), comprising 10 items across two subdimensions: cognitive reappraisal (CR1–CR6) and expressive suppression (ES7–ES10). Responses were rated on a 7-point Likert scale (1 = strongly disagree to 7 = strongly agree). Reliability in this sample was satisfactory, with α = 0.879 for cognitive reappraisal and α = 0.845 for expressive suppression. Representative items included: “I try to reinterpret situations before my emotions intensify” (cognitive reappraisal) and “I control my facial expressions so others cannot see when I am upset” (expressive suppression).

To evaluate Teacher Self-Efficacy (TSE), the Teachers’ Sense of Efficacy Scale (TSES) developed by Tschannen-Moran and Hoy (2001) was utilized. A shortened version was used for this study, consisting of 12 items evenly distributed across three dimensions: efficacy in student engagement (TSES1–TSES4), efficacy in instructional strategies (TSES5–TSES8), and efficacy in classroom management (TSES9–TSES12). Items were rated on a 9-point Likert scale (1 = not at all capable to 9 = highly capable). Cronbach’s α values demonstrated good reliability: student engagement (α = 0.849), instructional strategies (α = 0.862), and classroom management (α = 0.842). Representative items included: “Your ability to motivate students with low interest in learning,” “Your ability to adjust instructional strategies according to students’ needs,” and “Your ability to maintain students’ attention and order in the classroom.”

### 3.3 Data collection

Data were collected using an online survey platform. At Time 1 (T1), psychological capital (PsyCap), emotion regulation (ER), and teacher self-efficacy (TSE) were assessed, and 606 valid responses were obtained for the cross-sectional analyses. For the longitudinal study, the same cohort was followed across two additional waves. At Time 2 (T2), conducted 3 months after T1, ER was reassessed; at Time 3 (T3), conducted 3 months after T2, TSE was reassessed. The three-month interval between waves was selected to balance theoretical sensitivity and practical feasibility. Methodological work on longitudinal mediation emphasizes that observed effects may depend on the chosen lag length and that the optimal interval should align with the expected time scale of change in the constructs of interest (Cole and Maxwell, 2003; Dormann and Griffin, 2015; Maxwell and Cole, 2007). Given the practical constraints of school-based longitudinal data collection and the aim of capturing short-to-medium temporal ordering among psychological capital, emotion regulation, and teacher self-efficacy, a three-month lag was deemed appropriate. A total of 412 teachers completed all three waves and constituted the longitudinal sample used for the time-lagged mediation analyses. All participants provided informed consent, and the study protocol was approved by the institutional ethics committee. Descriptive statistics and ANOVA were conducted using SPSS 23.0; CFA and SEM were performed using AMOS 24.0.

### 3.4 Data analysis

Data analysis followed a theory-driven, two-stage analytic strategy. Prior to hypothesis testing, data were screened for distributional assumptions. Univariate normality was assessed via skewness and kurtosis, all of which fell within acceptable ranges (±2). Variance inflation factors indicated no evidence of problematic multicollinearity. Multivariate outliers were screened using Mahalanobis distance (df = number of observed indicators). Cases exceeding the chi-square critical value at *p* < 0.001 were flagged for inspection; after reviewing response patterns and influence diagnostics, they were retained because they did not materially affect model estimates. For the longitudinal analyses, missing data were handled using full information maximum likelihood (FIML), which allows for unbiased parameter estimation under the assumption of missing at random.

In the first stage, confirmatory factor analysis (CFA) was conducted using the baseline sample (*N* = 606) to establish the measurement reliability and construct validity of psychological capital (PsyCap), emotion regulation (ER), and teacher self-efficacy (TSE). In the second stage, structural equation modeling (SEM) was employed to test the hypothesized relationships among PsyCap, ER, and TSE. Cross-sectional SEM analyses were first performed using Time 1 data to examine the concurrent mediation structure.

Longitudinal hypotheses were subsequently examined using a three-wave panel subsample (*N* = 412). A time-lagged mediation model was specified in which PsyCap measured at Time 1 predicted ER at Time 2, which in turn predicted TSE at Time 3. This temporal specification reflects the proposed resource–regulation–belief ordering and focuses on prospective associations across measurement waves. Given the available measurement occasions, a parsimonious time-lagged mediation model was estimated rather than a full cross-lagged panel model. All analyses were conducted using SPSS 23.0 and AMOS 24.0. Indirect effects were tested using bias-corrected bootstrapping with 5,000 resamples.

## 4 Results

One-way analyses of variance (ANOVA) revealed systematic differences across key demographic and professional groups. Age-related differences were observed for psychological capital (PsyCap) and teacher self-efficacy (TSE), including all respective subdimensions (*p* < 0.01). Teachers aged 50 years and above consistently reported the highest levels of psychological resources and efficacy beliefs, followed by those aged 40–49 years, whereas younger teachers scored significantly lower. This pattern suggests that psychological capital and efficacy beliefs tend to strengthen with accumulated professional experience.

Significant differences were also found across professional title groups (*p* < 0.001). Teachers holding senior or advanced titles reported higher levels of PsyCap, emotion regulation (ER), and TSE than teachers of lower rank, indicating that professional seniority is associated with stronger psychological resources, greater regulatory capacity, and more robust instructional beliefs.

Differences related to administrative roles and school context further supported this trend. Teachers serving in school-level or middle management positions demonstrated higher teacher self-efficacy—particularly in student engagement—than homeroom and non-administrative teachers (*p* < 0.05). In addition, teachers from different school locations differed significantly on several indicators of self-efficacy and emotion regulation (*p* < 0.05). Taken together, these findings suggest that age, professional seniority, leadership experience, and school context constitute important background factors shaping teachers’ psychological resources, emotion regulation competence, and efficacy beliefs in Chinese primary education.

Table 2 presents the descriptive statistics, reliability, and validity indices for psychological capital (PsyCap), emotion regulation (ER), and teacher self-efficacy (TSE) among 606 Chinese primary school teachers. Teachers reported moderately high levels of PsyCap (*M* = 4.15, SD = 1.00) and ER (*M* = 4.89, SD = 1.16), whereas TSE (*M* = 5.94, SD = 1.48) showed the highest mean, indicating generally strong self-beliefs. Given the observed standard deviations (SD_TSE = 1.48; SD_PsyCap = 1.00), teacher self-efficacy exhibited the greatest dispersion, whereas psychological capital showed the least, indicating greater individual variability in efficacy beliefs. All skewness and kurtosis values were within ±2, confirming normality. PsyCap (Sk = −0.77, Ku = −0.60) and TSE (Sk = −1.00, Ku = −0.06) showed mild negative skew and near-normal distributions, whereas ER (Sk = −1.07, Ku = 0.21) was slightly more left-skewed, implying overall high and homogeneous emotion-regulation ability.

**Table 2:** Descriptive statistics, correlations, and construct validity (*N* = 606).

Variable	*M*	SD	Skew	Kurt	CR	AVE	MSV	MaxR(H)	1	2	3
1. PsyCap	4.15	1.00	−0.77	−0.60	0.94	0.70	0.436	0.925	1		
2. ER	4.89	1.16	−1.07	0.21	0.88	0.56	0.306	0.894	0.521**	1	
3. TSE	5.94	1.48	−1.00	−0.06	0.87	0.59	0.445	0.879	0.660**	0.553**	1

***p < 0.001; **p < 0.01.

The measurement model exhibited strong psychometric properties. Composite reliability (CR = 0.94, 0.88, 0.87) exceeded 0.70, and average variance extracted (AVE = 0.70, 0.56, 0.59) surpassed 0.50, indicating good internal consistency and convergent validity. Maximum shared variance (MSV = 0.31–0.45) was below corresponding AVE values, confirming discriminant validity. MaxR(H) coefficients (0.925, 0.894, 0.879) further supported construct reliability. All inter-construct correlations were positive and significant (*p* < 0.01): PsyCap correlated with ER (*r* = 0.52) and TSE (*r* = 0.66), and ER correlated with TSE (*r* = 0.55). These results suggest that teachers with higher psychological capital tend to regulate emotions more effectively and hold stronger instructional efficacy beliefs. Overall, the measurement model demonstrated excellent reliability and validity, providing a solid basis for subsequent SEM analyses.

Given the moderate to strong correlations among psychological capital (PsyCap), emotion regulation (ER), and teacher self-efficacy (TSE), it is important to clarify their conceptual and empirical distinctiveness. Conceptually, PsyCap represents a higher-order positive psychological resource encompassing hope, optimism, resilience, and general self-efficacy, whereas ER refers to regulatory processes governing emotional responses, and TSE reflects a domain-specific belief regarding instructional capability. Thus, the three constructs capture related but theoretically separable aspects of teachers’ psychological functioning.

Empirically, discriminant validity was supported through confirmatory factor analysis. For all constructs, average variance extracted (AVE) exceeded the recommended threshold of 0.50, and the square roots of AVE were greater than the corresponding inter-construct correlations. In addition, maximum shared variance (MSV) values were consistently lower than AVE values, indicating that shared variance between constructs did not exceed variance explained by their indicators. These results suggest that PsyCap, ER, and TSE are empirically distinguishable rather than reflections of a single latent factor.

To further reduce the risk of spurious associations and Type I statistical error, mediation effects were tested using bias-corrected bootstrapping with 5,000 resamples, which does not rely on normality assumptions and provides more conservative confidence intervals. Moreover, although self-efficacy is included as a subdimension of PsyCap, it reflects a generalized psychological resource, whereas TSE represents task- and context-specific instructional beliefs; consistent with prior research, these constructs were therefore retained and modeled as theoretically related but non-redundant. Taken together, these analytical safeguards enhance confidence that the observed relationships reflect substantive mechanisms rather than measurement overlap or statistical artifacts.

Table 3 presents the goodness-of-fit indices for the hypothesized structural equation model (SEM). The results indicate that the proposed structural model demonstrates an acceptable fit to the data. The ratio of chi-square to degrees of freedom (χ^2^/df = 1.509) is well below the recommended threshold of 3.0, indicating an excellent overall model fit. Both the Goodness-of-Fit Index (GFI = 0.913) and the Adjusted Goodness-of-Fit Index (AGFI = 0.901) exceed the conventional cutoff of 0.90, indicating that the model adequately fits the observed data. Furthermore, the Comparative Fit Index (CFI = 0.972), Tucker–Lewis Index (TLI = 0.970), and Incremental Fit Index (IFI = 0.972) all exceed the 0.95 criterion, demonstrating superior fit relative to the baseline model. The Normed Fit Index (NFI = 0.923) also exceeds 0.90, further supporting the model’s robustness. The Root Mean Square Error of Approximation (RMSEA = 0.029) is below the stringent cutoff of 0.05, indicating a minimal discrepancy between the implied and observed covariance matrices and confirming an excellent fit. Taken together, all indices meet or exceed the “excellent” or “acceptable” benchmarks, suggesting that the proposed structural model fits the data well and effectively captures the latent relationships among the constructs.

**Table 3:** Goodness-of-fit indices for the structural equation model.

Fit index	Recommended threshold	Obtained value	Evaluation
χ^2^/df	<3.00	1.509	Excellent
GFI	>0.90	0.913	Acceptable
AGFI	>0.90	0.901	Acceptable
CFI	>0.90	0.972	Excellent
TLI	>0.90	0.970	Excellent
IFI	>0.90	0.972	Excellent
NFI	>0.90	0.923	Acceptable
RMSEA	<0.08 (Good < 0.05)	0.029	Excellent

**Figure 1:**
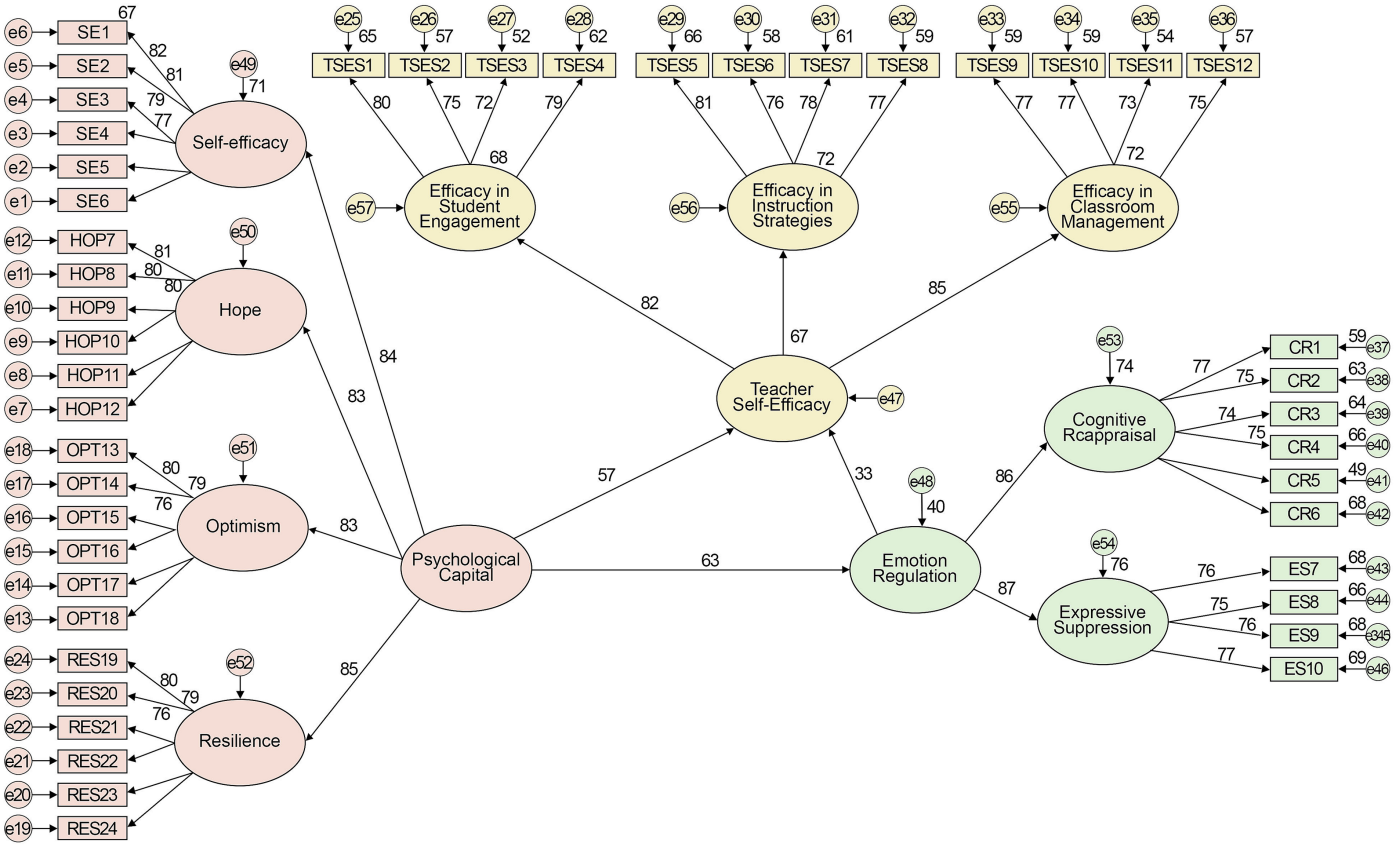
Cross-sectional structural equation model with standardized path coefficients.

Figure 1 presents the results of the structural equation modeling (SEM) analysis. Psychological capital exerted a significant positive effect on emotion regulation (β = 0.631, *p* < 0.001), indicating that teachers with higher levels of psychological resources—namely hope, optimism, resilience, and self-efficacy—are more likely to adopt adaptive emotion-regulation strategies. Moreover, psychological capital had a significant direct effect on teacher self-efficacy (β = 0.566, *p* < 0.001), suggesting that teachers with richer psychological resources tend to exhibit stronger confidence and a greater sense of control in their instructional practice. In addition, emotion regulation was positively associated with teacher self-efficacy (β = 0.333, *p* < 0.001), implying that teachers who can effectively manage their emotions perceive themselves as more competent and efficacious in teaching.

The findings provide empirical support for the hypothesized mediation model and identify emotion regulation as a critical link between teachers’ psychological capital and self-efficacy. Results from the bootstrapping mediation analysis (5,000 resamples) demonstrate that psychological capital significantly influences teacher self-efficacy (β = 0.776, 95% CI [0.718, 0.827], *p* < 0.001), with a notable direct effect (β = 0.566, 95% CI [0.465, 0.663], *p* < 0.001). Emotion regulation exhibited a significant partial mediating effect (β = 0.210, 95% CI [0.138, 0.290], *p* < 0.001), indicating that emotional strategies serve as a primary mechanism by which psychological capital enhances self-efficacy. Table 4 presents the detailed results of the mediation analysis.

**Table 4:** Direct, indirect, and total effects.

Effect type	Estimate	95% CI Lower	95% CI Upper	*p*-value
Direct effect	0.566	0.465	0.663	<0.001
Indirect effect	0.210	0.138	0.290	<0.001
Total effect	0.776	0.718	0.827	<0.001

**Figure 2:**
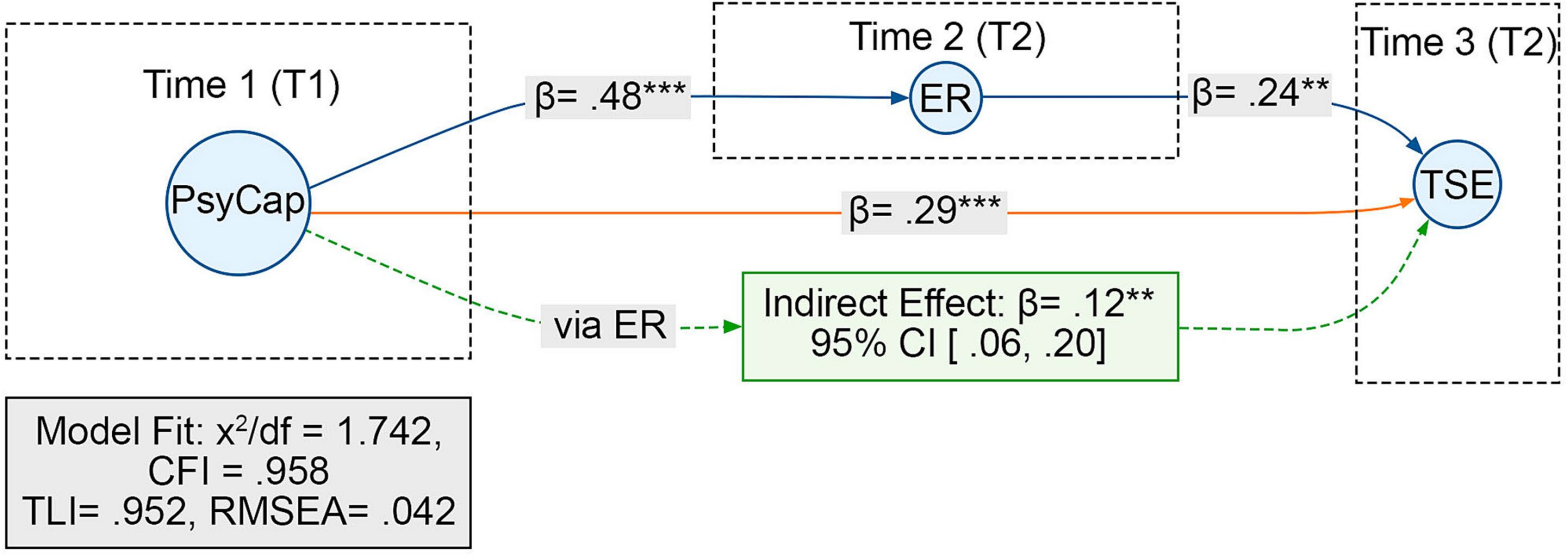
Three-wave time-lagged mediation model linking T1 PsyCap, T2 ER, and T3 TSE. ***p < 0.001; **p < 0.01.

The three-wave time-lagged mediation model demonstrated satisfactory model fit (χ^2^/df = 1.742, CFI = 0.958, TLI = 0.952, RMSEA = 0.042). Table 5 summarizes the standardized path coefficients and confidence intervals for the longitudinal mediation model. In the three-wave time-lagged mediation model, PsyCap at T1 significantly predicted ER at T2 (β = 0.48, *p* < 0.001) and TSE at T3 (β = 0.29, *p* < 0.001). In addition, ER at T2 significantly predicted TSE at T3 (β = 0.24, *p* < 0.01), providing longitudinal support for ER as an intervening mechanism. The indirect longitudinal effect of PsyCap_T1 on TSE_T3 via ER_T2 was significant (β = 0.12, *p* < 0.01; 95% CI [0.06, 0.20]), accounting for approximately 29% of the total longitudinal association. Figure 2 illustrates the corresponding three-wave time-lagged mediation structure linking PsyCap, ER, and TSE across time. These findings provide temporal support for a resource–regulation–belief pathway, whereby teachers’ psychological capital initiates adaptive emotion regulation that fosters stronger efficacy beliefs over time.

**Table 5:** Longitudinal structural model results (*N* = 412).

Pathway	β	*p*	95% CI (Lower–Upper)
PsyCap_T1 → ER_T2	0.48	<0.001	[0.36, 0.60]
ER_T2 → TSE_T3	0.24	<0.01	[0.11, 0.37]
PsyCap_T1 → TSE_T3	0.29	<0.001	[0.18, 0.41]
Indirect (T1 → T2 → T3)	0.12	<0.01	[0.06, 0.20]

## 5 Discussion

This study examined a resource–regulation–belief framework connecting psychological capital (PsyCap), emotion regulation (ER), and teacher self-efficacy (TSE) among Chinese primary school teachers. Structural equation modeling revealed three key findings. First, PsyCap positively predicted ER, suggesting that teachers with higher hope, optimism, resilience, and generalized self-efficacy are more likely to adopt adaptive emotion-regulation strategies (Bing et al., 2022; Gross, 1998). Second, PsyCap directly predicted TSE, supporting the view that psychological resources strengthen efficacy beliefs (Avey et al., 2011; Bandura, 1997; Luthans et al., 2007). Third, ER positively predicted TSE and partially mediated the PsyCap–TSE relationship, accounting for 27% of the total effect (Burić and Macuka, 2018). Longitudinal analyses further indicated that PsyCap predicted later ER and TSE, providing temporal support for its role as an upstream psychological resource that initiates adaptive regulation and contributes to teachers’ subsequent efficacy beliefs.

Beyond replicating prior findings on the positive associations among psychological capital, emotion regulation, and teacher self-efficacy, the present study extends existing literature in several important ways. First, the associations among psychological capital, emotion regulation, and teacher self-efficacy observed in the present study are consistent with prior cross-sectional and longitudinal research indicating that psychological resources are linked to adaptive regulation processes and stronger efficacy beliefs (Burić and Macuka, 2018; Ma, 2023), while further situating these relationships within a unified resource–regulation–belief framework. Second, compared with studies conducted in Western contexts, the relatively strong role of emotion regulation observed in this Chinese primary-school sample may reflect the high emotional labor, hierarchical organizational structures, and strong normative expectations placed on teachers in collectivist educational settings (Day and Gu, 2013; Yin et al., 2016). In such contexts, teachers’ capacity to regulate emotions may be especially critical for transforming internal psychological resources into sustained instructional confidence.

Methodologically, by combining cross-sectional SEM with a three-wave time-lagged mediation model, this study advances prior research that has largely relied on single-wave designs. Although causal inference remains cautious, the temporal ordering of PsyCap, ER, and TSE provides stronger support for the proposed mechanism than cross-sectional evidence alone.

These findings align with prior research demonstrating that TSE underpins classroom processes and teacher well-being (Tschannen-Moran and Hoy, 2001), and that ER supports effective functioning under emotional demands (Yin et al., 2016). By integrating Luthans’ positive psychological capital theory with Gross’s process model of ER, the present study illustrates how PsyCap not only buffers stress but also generates proactive, antecedent-focused strategies that reinforce efficacy beliefs (Luthans et al., 2006; Sweetman and Luthans, 2010). The observed temporal pattern among psychological capital, emotion regulation, and teacher self-efficacy suggests that teachers’ psychological resources function as foundational elements in professional development, rather than merely short-term outcomes of motivational states. In the Chinese primary education context, characterized by high emotional labor, hierarchical structures, and collective norms, PsyCap appears particularly critical for maintaining self-efficacy and emotional resilience (Day and Gu, 2013; Klassen and Chiu, 2010). Practically, the findings highlight a dual-track professional development approach: (1) PsyCap empowerment through goal-setting and pathway training (hope), attribution retraining and learned optimism (optimism), mastery modeling (self-efficacy), and resilience-building programs (resilience) (Luthans et al., 2006). (2) ER skill training emphasizing antecedent-focused strategies—such as cognitive reappraisal, situation modification, and attentional deployment—while reducing dependence on suppression (Gross, 1998; Hulsheger et al., 2013).

School-level systems including mentoring, psychologically safe climates, and recognition of effort and growth can amplify these effects (Collie et al., 2012; Jennings and Greenberg, 2009). In the era of digital education, emerging AI-supported tools may further facilitate reflective emotion regulation and strengthen the connections between teachers’ psychological resources, regulatory processes, and efficacy beliefs (Celik et al., 2022; Holmes et al., 2019). Attention, however, should be given to technostress and digital inequality to ensure that technology enhances rather than erodes teachers’ psychological resources (Lin et al., 2023; Yang and Du, 2024).

Several limitations warrant caution. Although the longitudinal design provides temporal validation, causal inference remains constrained, and unmeasured contextual factors could jointly affect PsyCap, ER, and TSE (Hamaker et al., 2015; Ilies et al., 2007). Future research could adopt multi-wave, multi-level, or intervention designs to test causal directions and contextual moderators. Cross-cultural comparisons within the Asia-Pacific region would further clarify the boundary conditions of the resource–regulation–belief mechanism across diverse educational systems. In summary, this study offers evidence that teachers’ psychological resources are transformed into stronger efficacy beliefs through emotion regulation. Integrating PsyCap cultivation with ER-focused strategy training and embedding these within supportive organizational contexts may sustainably enhance teachers’ instructional confidence and well-being, thereby improving students’ learning experiences.

## 6 Conclusion and implications

This study validates a resource–regulation–belief framework among Chinese primary school teachers, demonstrating that psychological capital (PsyCap) enhances teacher self-efficacy (TSE) directly and indirectly through emotion regulation (ER). Teachers with higher levels of hope, optimism, resilience, and self-efficacy are more likely to engage in adaptive emotion-regulation strategies—particularly cognitive reappraisal—when facing classroom stress. Over time, these regulatory processes help sustain and strengthen teachers’ instructional confidence. Longitudinal evidence from the present study indicates that psychological capital operates through emotion regulation to support the development of teacher self-efficacy, highlighting a process through which psychological resources buffer stress and facilitate efficacy growth. Grounded in Luthans’ PsyCap theory and Gross’s process model of emotion regulation, these findings extend positive psychology into the Chinese context, where teachers often face high emotional labor and hierarchical work structures (Erden, 2025).

Practically, teacher development should integrate PsyCap enhancement and ER skill training. Effective modules include goal-setting and pathway planning (hope), attribution retraining and learned optimism (optimism), mastery modeling and resilience-building (resilience), and cognitive reappraisal workshops (ER) (Bertieaux et al., 2024; Liu and Du, 2024). Supportive school climates that promote collegial trust and professional growth further sustain teachers’ emotional well-being and instructional quality (Collie et al., 2012; Jennings and Greenberg, 2009).

In AI-supported classrooms, intelligent dashboards and tutoring systems can serve as emotional scaffolds that reduce cognitive load and enhance self-reflection (Chen et al., 2024; Duan and Zhao, 2024; Oh and Ahn, 2024). Future longitudinal and mixed-method research should explore how psychological capital shapes teachers’ emotion-regulation processes and, in turn, their self-efficacy as these relationships unfold within contexts of digital transformation, thereby generating scalable evidence to inform teacher development across the Asia-Pacific region.

## 7 Limitations and future directions

Several limitations should be acknowledged. Although the three-wave longitudinal design represents a methodological strength, causal inferences remain limited. Unmeasured contextual factors—such as teacher burnout, school climate, leadership support, or student characteristics—may simultaneously influence psychological capital, emotion regulation, and teacher self-efficacy. Future studies could incorporate these variables within multi-level or integrative models to more comprehensively capture the contextual conditions under which psychological resources are translated into efficacy beliefs.

In addition, the three-month intervals between measurement waves, while consistent with prior longitudinal studies, may not fully capture longer-term developmental changes in teachers’ emotion regulation and efficacy beliefs. Future research could enhance temporal precision by increasing the number of measurement waves, standardizing or varying time lags, and implementing strategies to reduce participant attrition. Moreover, intervention-based or quasi-experimental designs, as well as multi-level modeling approaches, would help clarify causal pathways and examine how individual psychological resources interact with school-level conditions over time.

## Data Availability

The datasets presented in this study can be found in online repositories. The names of the repository/repositories and accession number(s) can be found in the article/Supplementary material.
